# Normalizing the Tumor Microenvironment: A New Frontier in Ovarian Cancer Therapy

**DOI:** 10.3390/ijms27020939

**Published:** 2026-01-17

**Authors:** Adam P. Jones, Yanxia Zhao, Bo R. Rueda, Oladapo O. Yeku, Lei Xu

**Affiliations:** 1 Edwin L. Steele Laboratories, Department of Radiation Oncology, Massachusetts General Hospital, Harvard Medical School, Boston, MA 02114, USA; 2 Cancer Center, Union Hospital, Tongji Medical College, Huazhong University of Science and Technology, Wuhan 430023, China; 3 Vincent Center for Reproductive Biology, Department of Obstetrics and Gynecology, Massachusetts General Hospital, Harvard Medical School, Boston, MA 02114, USA; brueda@mgh.harvard.edu; 4 Gynecologic Cancers Program, Massachusetts General Hospital, Harvard Medical School, Boston, MA 02114, USA

**Keywords:** ovarian cancer, tumor microenvironment, extracellular matrix, metastasis, immunotherapy

## Abstract

Ovarian cancer is one of the deadliest gynecological malignancies, where most patients become clinically symptomatic at advanced stages of disease due to the lack of effective diagnostic screening. Despite recent advances in surgical resection and chemotherapy, recurrent ovarian cancer remains largely refractory to treatment, resulting in poor prognosis. The ovarian cancer tumor microenvironment (TME) is highly abnormal and presents a significant barrier to successful therapy. A combination of abnormal vasculature, desmoplastic extracellular matrix, and aberrantly activated hypoxic and immune-suppressive pathways culminates in promoting tumor growth, dissemination, chemoresistance, and immunosuppression. Whilst immune checkpoint inhibitors have shown success in other cancers, their application in ovarian cancer, particularly at advanced stages, remains limited. In this review, we discussed the application of tumor extracellular matrix normalizing therapies in preclinical models of advanced ovarian cancer, and their synergistic benefit to chemotherapy and immunotherapy. Collectively, these insights underscore TME normalization as a promising therapeutic strategy with the potential to improve ovarian cancer management.

## 1. Introduction

Ovarian cancer (OC) is among the most prevalent and lethal gynecologic malignancies, with an estimated 20,890 new cases and 12,730 deaths in the United States in 2025 [1]. Despite advances in surgical techniques and systemic therapy, OC continues to be one of the leading causes of gynecologic oncology-related mortality. The high lethality is largely attributed to the difficulty of early detection, given the absence of specific symptoms and reliable screening strategies, which results in most patients being diagnosed at a late-stage [2,3]. Late-stage diagnosis is frequently accompanied by widespread peritoneal dissemination, high tumor burden, and poor prognostic outcomes. The current standard treatment consists of maximal cytoreductive surgery followed by a chemotherapy regimen that includes a platinum complex (carboplatin or cisplatin) and a taxane (paclitaxel or docetaxel). Patients with BRCA1/2 mutations or homologous recombination-deficient (HRD) tumors often receive poly ADP-ribose polymerase (PARP) inhibitor-based maintenance therapy to prolong progression-free survival [4]. Whilst patients exhibit initial responsiveness, the majority will experience relapse, with recurrent tumors resistant to chemotherapy [5,6]. Treatment resistance presents the primary barrier to long-term survival.

The tumor microenvironment (TME) is a complex and dynamic ecosystem that plays a critical role in tumor progression, metastatic dissemination, and treatment resistance [7,8]. Unlike normal tissue, the TME is profoundly abnormal, characterized by structural, cellular, and molecular alterations that support tumor growth and survival. Tumor vasculature is structurally and functionally abnormal, exhibiting chaotic architecture, irregular blood flow, and increased permeability [9]. These characteristics not only impair efficient oxygen and nutrient delivery, fostering hypoxia, but also create gradients that drive invasive behavior and metastasis [10]. Furthermore, the abnormal vasculature limits the delivery of therapeutic agents, reducing treatment efficacy [11]. Immune suppression within the TME is another hallmark feature. Tumors upregulate immune regulatory pathways to evade immune system detection and elimination. This is achieved by recruiting immunosuppressive cell populations, such as regulatory T cells (Tregs) and myeloid-derived suppressor cells (MDSCs), and by the expression of immune checkpoint molecules. These mechanisms create an immunosuppressive milieu that not only supports tumor survival but also undermines the efficacy of immunotherapy [12].

Major strides have been made in deciphering how dysregulated extracellular matrix (ECM) deposition and remodeling reshape the TME and contribute to a pro-tumorigenic, immunosuppressive desmoplastic environment. Excessive ECM deposition and stiffening facilitate cancer cell invasion by providing physical pathways for migration and promoting survival signals. The altered ECM also acts as a barrier to therapeutic agent penetration, further compounding treatment resistance [13,14]. Together, these pro-tumorigenic features advance cancer progression and limit treatment efficacy, highlighting the urgent need for novel interventions.

The renin–angiotensin system (RAS) is recognized for its critical role in maintaining cardiovascular homeostasis and regulating fluid and electrolyte balance. Although initially identified as a vasoconstrictor, angiotensin II (AngII) is also recognized for its involvement in ECM formation [15]. Among all FDA-approved antihypertensive Angiotensin II type I receptor blockers (ARBs), losartan exhibits optimal kinetics for tissue penetration and distribution [16,17,18]. Studies in mouse models of breast and pancreatic cancer showed that losartan reduces intratumoral expression of thrombospondin-1 (THBS-1), an activator of transforming growth factor beta (TGF-β), resulting in a significant decrease in intratumoral collagen and hyaluronan. This reduction in solid stress and vascular compression correlated with improved vascular perfusion, resulting in decreased tumor hypoxia and improved delivery and efficacy of both small-molecule drugs and nanomedicine [19,20]. These findings paved the way for a successful Phase II trial of losartan in combination with FOLFIRINOX and radiotherapy in locally advanced pancreatic cancer patients, where downregulation of pro-invasion and immunosuppression-related genes was observed and correlated with improved overall survival [21]. The Phase II trial of neoadjuvant FOLFIRINOX plus losartan followed by chemoradiotherapy in locally advanced pancreatic cancer achieved a median overall survival of 31.4 months and an R0 resection rate of 61%, substantially outperforming historical outcomes in this setting [22].

In OC, several important findings have emerged: first, there is an inverse correlation between tumor fibrosis levels and both recurrence-free and overall survival [23]; second, serum levels of angiotensin-converting enzyme (ACE), which is responsible for converting inactive angiotensin I (AT1) to the biologically active AngII, are increased [24]; and third, high AT1 expression levels are associated with adverse patient outcomes. The potential of losartan to modulate the tumor microenvironment and enhance chemotherapeutic efficacy in OC, however, has only been explored in preclinical models of HGSOC and retrospective analyses of clinical patient data so far [25]. Further studies are required to understand how patient heterogeneity may influence the clinical utility of losartan and to identify responsive biomarkers for effective treatment monitoring. In this review, we explore the role of ECM in ovarian cancer progression, metastasis, and treatment resistance, and the potential of ECM-targeted therapies to improve outcomes in patients with OC.

## 2. The Composition and Function of the Extracellular Matrix on Ovarian Cancer Development, Metastasis, and Chemoresistance

Whilst the cellular basis of ovarian tumorigenesis has dominated the field, there is growing appreciation of the acellular environment, which has profound implications for cellular communication, survival, migration, and metastasis in OC. Initial studies utilizing Second-Harmonic Generation revealed that malignant ovaries were less cellular and rich in newly synthesized collagens [26]. Transcriptomic studies have provided greater depth to these findings, showing that metastatic lesions from high-grade serous ovarian carcinomas (HGSOC) were enriched for fibrotic ECM genes compared with the primary tumor. Furthermore, these changes are amplified by platinum chemotherapy, particularly with respect to collagen 6 (*COL6*), which has been associated with enhanced resistance [27]. Analysis of 36 HGSOCs indicated that ECM-related genes were further upregulated in patients with advanced progression and poor prognosis, including *COL1A1*, *COL11A1*, cartilage oligomeric matrix protein (*COMP*), versican (*VCAN*), fibronectin-1 (*FN1*), and cathepsin B (*CTSB*) [28]. Hence, changes in ECM are core drivers of OC evolution and aggressiveness. Importantly, these ECM-driven effects can be broadly categorized into (i) biomechanical mechanisms, such as matrix stiffening, solid stress, and physical barrier formation, and (ii) biochemical and signaling mechanisms, including integrin-mediated adhesion, growth factor presentation, and activation of pro-survival and stemness pathways. These mechanisms operate across distinct compartments of the tumor microenvironment, including tumor cells, the vasculature, immune infiltrates, and cancer stem cell (CSC) niches, and are differentially targetable by emerging therapeutic strategies.

An important metastatic site in patients with OC is the peritoneal cavity, driven mainly by the generation of multicellular tumor spheroids from the primary lesion that disseminate via ascites [29]. Typically, anchorage-dependent cells detaching from the ECM would undergo anoikis, a form of programmed cell death [30]; however, by detaching collectively, these tumorspheres bypass this cell death mechanism, allowing tumor cells to survive and disseminate. Interestingly, these tumorsphere seed metastatic tumors with the same intra-tumoral heterogeneity as the primary tumor [31]. Tumorsphere development depends on L1 cell adhesion molecule (L1CAM)-mediated upregulation of a5b1 integrins and fibronectin binding, as blocking b1 integrins abrogates tumorsphere development [32,33]. Further studies in HSGOC patients have shown that platelet-derived growth factor receptor beta (PDGFRβ) is essential for fibronectin-mediated clustering in malignant ascites [34], and that ECM production after initial detachment supports metastatic spread [35]. Here, ECM remodeling contributes both mechanically, by enabling collective detachment and protection from anoikis through multicellular aggregation, and biochemically, by engaging integrin- and PDGFRβ-dependent signaling pathways that actively promote tumorsphere survival, cohesion, and metastatic competence. While tumorsphere formation supports survival during peritoneal transit, successful metastatic colonization requires subsequent attachment to mesothelium, invasion, and expansion within secondary niches. These later steps remain highly dependent on ECM density, stiffness, and ligand availability, suggesting that normalization strategies that reduce matrix deposition and mechanical stress may preferentially disrupt spheroid anchoring and early outgrowth without necessarily affecting initial dissemination. Notably, the efficacy of such approaches is likely influenced by the degree of desmoplasia, ascites composition, and stromal activation within the peritoneal cavity, which differ markedly between patients and may evolve during disease progression or following chemotherapy.

ECM plays a pivotal role in the morphodynamic changes that underlie tumor-spheroid induction, maintenance, and invasion into the peritoneal cavity during OC pathogenesis. Firstly, OC tumor spherioids undergo epithelial-to-mesenchymal transition (EMT), driven by stimuli such as TGF-β in malignant ascites, with changes in ECM composition, including E-cadherin downregulation and fibronectin upregulation, thereby facilitating their invasiveness [36]. EMT enables tumor spheroids to become anoikis-resistant and promotes mesothelial attachment through the fibronectin receptor [37,38]. Upon mesothelial invasion, OC cells undergo the reverse process of mesenchymal-to-epithelial transition (MET), thereby enabling the establishment of omental and peritoneal metastases, characterized by downregulation of fibronectin [39,40]. Upon colonization of the metastatic niche, OC cells begin to proliferate through ECM interactions. Collagen I and heparin-binding epidermal growth factor (EGF) produced by omental fibroblasts create a proliferative environment to stimulate OC growth [41]. Adipose tissue also plays an interesting role in OC metastasis, whereby the desmoplastic evolution within the omentum is driven by adipocyte-derived interleukin (IL)-8 and enables metastatic spread [42]. A recent study has also shown that bidirectional extracellular signaling between adipocyte-derived mesenchymal stem cells and OC cells stimulates ECM remodeling, EMT plasticity, and metastatic behavior [43]. This communication is mediated by small extracellular vesicles and upregulates pro-tumorigenic pathways such as TGF-β/Smad and Wnt/β-catenin, and, through in silico analyses, has been shown to be associated with poor prognostic outcomes in patients with OC. Notably, these processes reflect a coordinated interplay between ECM structural remodeling, which alters tissue architecture and mechanical permissiveness for invasion, and ECM-mediated signaling, which drives EMT/MET plasticity through TGF-β, Wnt/β-catenin, and integrin-dependent pathways. Distinguishing these roles is essential, as mechanical normalization strategies primarily affect tissue stiffness and interstitial pressure, whereas signaling-targeted approaches modulate transcriptional programs governing invasion and stemness. Importantly, this distinction also defines therapeutic windows during metastatic progression: mechanical normalization is predicted to impair mesothelial clearance and early spheroid engraftment by reducing permissive tissue compliance, whereas signaling-based interventions more directly suppress EMT-driven invasion and stem-like plasticity required for durable metastatic colonization. At the same time, targeting upstream regulators such as TGF-β presents translational challenges due to its pleiotropic and context-dependent roles in tissue homeostasis, epithelial integrity, and immune regulation, raising concerns regarding systemic toxicity and unintended biological consequences.

Evidence of ECM-driven proliferation of OC cells was initially shown in various in vitro cell culture experiments using Hey-A8, OVCAR-3 and Peo.36 cell lines, all of which laminin is overexpressed in OC compared to healthy tissues, particularly the variant laminin a5, which is associated with poor prognosis; genetic knockdown of *LAMA5* was shown to inhibit tumor growth and metastasis in vivo by attenuating Notch signaling [44]. Hyaluronic acid can also promote OC proliferation through CD44; 3D in vitro cultures have shown that mechanical strain interactions stimulate proliferation and resistance to paclitaxel [45]. These proliferative effects arise through both mechanotransduction, whereby matrix stiffness and strain activate intracellular sensors such as YAP/TAZ, and ligand–receptor signaling, including laminin-Notch and hyaluronan-CD44 interactions, highlighting multiple, non-redundant routes by which the ECM reinforces tumor growth.

Proteoglycans have also been shown to reinforce ECM-associated pro-tumorigenic signaling pathways and chemoresistance further. Among these, the chondroitin sulfate proteoglycan (CSPG) versican is consistently upregulated in ovarian tumors and metastatic lesions, where it associates with hyaluronan to form a highly hydrated pericellular matrix that promotes tumor cell survival, migration, and adhesion [46,47]. This versican–hyaluronan network enhances OC cell motility and peritoneal attachment via CD44-dependent signaling, facilitating transcoelomic dissemination while simultaneously providing protection from mechanical stress and anoikis [46,47,48]. Significantly, disruption of versican–hyaluronan interactions using hyaluronan oligosaccharides significantly reduces invasive behavior in OC models, underscoring the functional importance of this proteoglycan axis in disease progression [46]. Beyond metastasis, versican-rich matrices alter integrin and growth factor signaling, creating a microenvironment that favors cell survival and reduces sensitivity to cytotoxic therapies [27,48].

Heparan sulfate proteoglycans (HSPGs), including syndecans, glypicans, and perlecan, further contribute to chemoresistance by regulating growth factor bioavailability, cell–ECM adhesion, and drug penetration within the tumor microenvironment. Syndecan-1 expression is increased in ovarian tumors compared with normal ovarian tissue and has been associated with more aggressive disease phenotypes and altered responsiveness to therapy [49]. ECM-associated HSPGs, such as perlecan, bind and present pro-survival and pro-angiogenic growth factors, including FGF and VEGF, thereby reinforcing signaling pathways that promote tumor persistence under chemotherapeutic stress [50,51]. In addition, proteoglycan-rich ECM structures act as physical barriers that impair drug diffusion and sustain integrin-mediated survival signaling, a recognized mechanism of cell adhesion-mediated drug resistance in OC [27,52]. Enzymatic remodeling of proteoglycans, particularly by proteases such as a disintegrin and metalloproteinase with thrombospondin motifs 5 (ADAMTS5), has been shown to affect OC progression and treatment. ADAMTS5 is elevated in ovarian malignancies compared to borderline and benign lesions and is upregulated by OC cells overexpressing the small GTPase Rab25 [53,54]. Furthermore, ADAMTS5 expression is associated with advanced stages of OC and poor prognosis [53,55]. A recent study has shown that Rab25 upregulates ADAMTS5 in OC through NF-κB signaling and is necessary to stimulate OC cell invasiveness through 3D cancer-associated fibroblast (CAF) matrices, which was reduced by ADAMTS5 inhibition [53]. Collectively, these findings highlight proteoglycans as central drivers of ECM-mediated treatment resistance in ovarian cancer and support targeting them to disrupt tumor–stroma crosstalk.

The ECM plays a central role in shaping chemotherapeutic responses in OC. Progressive remodeling of the ECM—through altered composition, mechanical stiffening, and structural disorganization—occurs during tumor evolution, between primary and metastatic sites, and following chemotherapy exposure [27]. These dynamic changes create a protective microenvironment that enables tumor cell survival during platinum- and taxane-based treatments. Transcriptional profiling has repeatedly linked chemo-resistant phenotypes with increased expression of ECM and ECM-associated genes. In the A2780 OC cell line, resistance to cisplatin and paclitaxel correlates with upregulation of multiple collagen transcripts, including *COL6A3* in cisplatin-resistant variants [56]. Consistent with these findings, exposure of drug-sensitive cells to collagen VI is sufficient to induce cisplatin resistance in vitro [57]. Among collagen VI isoforms, *COL6A2* is significantly elevated in drug-resistant metastatic lesions and correlates with poor prognostic outcomes, whereas increased levels of *SERPINA10*, an ECM-remodeling regulator, are associated with platinum sensitivity [27,58]. ECM stiffening also enhances cell survival by reinforcing adhesion signaling. Inhibiting focal adhesion kinase (FAK) or Yes-associated protein (YAP,) a key mechanosensor, restores platinum sensitivity, highlighting the importance of adhesion-dependent resistance mechanisms [59]. This illustrates how ECM-induced mechanical stiffening is transduced into intracellular survival signaling via focal adhesions and mechanosensitive pathways, providing a mechanistic bridge between matrix biomechanics and tumor-intrinsic resistance programs. Notably, these same adhesion- and mechanotransduction-dependent survival pathways are engaged during spheroid reattachment and expansion at metastatic sites, indicating that ECM normalization may simultaneously limit chemoresistance and suppress the establishment of secondary lesions derived from disseminated spheroids.

Paclitaxel resistance is likewise linked to increased expression of collagens such as *COL1A2*, *COL4A1*, *COL16A1*, *COL17A1*, *COL18A1*, and *COL21A1*, as well as enhanced *LOX* expression, which promotes collagen crosslinking and matrix stiffening [56,60]. Interestingly, a new study showed the pan-lysyl oxidase (LOX) inhibitor PXS-5505 was able to reduce ECM stiffness in an HGSOC chemo-resistant murine model, and improve response to chemotherapy [61]. Collagen I modulates microtubule function by inducing nuclear tau protein, which competes with paclitaxel binding, thereby reducing drug efficacy [62,63,64]. Collagen I also diminishes cisplatin sensitivity by promoting β1-integrin-mediated adhesion and reducing cellular drug accumulation through decreased copper transporter 1 (CTR1) levels [27,65]. Despite these robust observations, some studies report no association between collagen I structure and paclitaxel resistance, underscoring context specificity in ECM–drug interactions [66].

Fibronectin also contributes significantly to chemoresistance. Preconditioning of OC cells with TGF-β1 enhances attachment to fibronectin, activating Akt signaling and reducing cisplatin-induced apoptosis [67]. Fibronectin similarly augments cisplatin resistance in HGSOC [27]. In taxane resistance, fibronectin promotes Akt2-dependent activation of survivin and suppresses p38/ASK1 apoptotic signaling, thereby protecting cells from cell death [68,69]. However, contradictory observations of reduced fibronectin mRNA in some paclitaxel-resistant ovarian endometrioid A2780 cell lines suggest that fibronectin-mediated mechanisms may vary between models [56]. Hyaluronic acid is also upregulated following carboplatin treatment and activates CD44 to induce ATP-binding cassette (ABC) transporter expression, enhancing drug efflux and correlating clinically with poor survival [70]. The hyaluronic acid-CD44 signaling axis also activates the embryonic stem cell transcription factor Nanog and, in conjunction with STAT3, promotes multi-drug resistance protein 1 (MDR1) expression, thereby enabling paclitaxel efflux. Nanog expression also upregulates markers of pluripotent stemness, including Rex1 and Sox2 [71]. Extracellular matrix protein-1 (ECM1) induces cisplatin resistance via integrin αXβ2 and downstream activation of Akt/FAK/Rho signaling, leading to ABC sub-family G member 1 (ABCG1)-mediated enhancement of cancer stem-like traits [72]. Consistent with this, ECM-mediated resistance frequently overlaps with cancer stem cell (CSC) biology. The CSC marker aldehyde dehydrogenase 1A1 (ALDH1A1) correlates with *COL1A2*, *LOX*, and *COL3A1* in paclitaxel-resistant cells and promotes the expression of major efflux pumps [73,74,75]. Pharmacologic inhibition of ALDH1A1 reduces transporter expression and re-sensitizes cells to chemotherapy [74]. Together, ECM-mediated chemoresistance in ovarian cancer reflects a complex interplay between matrix composition, biomechanical properties, tumor–stromal communication, and metabolic adaptation. Within this framework, CSC niches are particularly sensitive to ECM cues, as matrix stiffness, hyaluronan-rich pericellular coats, and integrin engagement cooperatively sustain stemness, drug efflux capacity, and quiescence. Thus, ECM remodeling not only protects bulk tumor cells but also preserves therapy-resistant CSC reservoirs.

## 3. Transforming Growth Factor (TGF)-β Inhibition for Ovarian Cancer

TGF-β is a pleiotropic growth factor that regulates cellular proliferation, differentiation, survival, and inflammatory pathways, and is often dysregulated in cancer [76]. In OC, TGF-β has been shown to be a driver of fibrotic responses, particularly collagen remodeling, and is associated with metastatic spread and poor prognosis [77]. To address the challenge of normalizing the fibrotic TME in OC, our lab initially explored the therapeutic potential of blocking TGF-β signaling in HGSOC OC models [78]. This study utilized a soluble TGF-β receptor II (sTβRII) to block TGF-β signaling in two xenograft OC models (SKOV3ip1 and Hey-A8). Inhibition of TGF-β signaling decreased vascular endothelial growth factor (VEGF) and IL-8 expression, key angiogenic and tumor growth factors in ovarian cancer [79,80], leading to reduced peritoneal tumor burden and restored diaphragm lymphatic vessel functionality, thereby reducing ascites accumulation. These findings highlight TGF-β’s dual role in regulating both angiogenesis and lymphangiogenesis and suggest TGF-β inhibition may interfere with metastatic spread to the peritoneum. Further studies have shown that TGF-β has complex roles at different stages of OC development and across different OC histopathological types, where TGF-β can induce apoptosis in ovarian surface epithelial cells and low-grade serous carcinomas, but can also stimulate invasiveness and EMT activation in xenografts of human serous borderline tumors and HGSOCs [81]. Together, these studies highlight the potential of TGF-β therapy for patients with OC. However, therapeutic responses to TGF-β blockade are likely to be heterogeneous, influenced by tumor subtype, disease stage, baseline immune contexture, and stromal composition, emphasizing the importance of identifying predictive biomarkers and defining patient populations most likely to benefit.

Various studies have shown that TGF-β induces chemoresistance to standard platinum therapies in many cancers, including OC, through EMT and stemness induction [82]. Recently, it was shown that chemoresistance is maintained by tumor hypoxia, whereby hypoxia stimulates TGF-β signaling, leading to hypoxia-inducible factor (HIF)-2a-regulated stemness in HGSOC [83]. Hence, targeting TGF-β to improve response to chemotherapy is of great interest. A study by Wang et al. showed that downregulation of TGF-β1 expression in the SKOV3 model inhibited tumor growth and increased chemosensitivity by upregulating BRCA1 and Smad3 phosphorylation [84]. Building on this, Newsted et al. developed antibodies against the Type II TGF-β receptor (TGFBR2) using phage display. These high-affinity antibodies were shown to potently suppress TGF-β signaling, EMT activation, and OC cell invasion in vitro, and improve chemotherapy response and limit immune exclusion in metastatic OC models [85].

TGF-β is not only a major driver of fibrotic responses in OC, but also a key mediator of immunosuppression and resistance to immunotherapies like immune checkpoint inhibitors (ICIs), with poor single-agent response rates around 10–25% in OC [86,87,88]. Multiple trials of PD-1/PD-L1 monotherapy, including JAVELIN, KEYNOTE-028, and KEYNOTE-100, reported only modest response rates with short progression-free survival (1.9–3.5 months), despite acceptable safety profiles [89,90,91]. These outcomes were not meaningfully improved by combining ICIs with chemotherapy or VEGF inhibition, as evidenced by large phase III trials such as JAVELIN-200 and IMagyn050, which failed to show a survival benefit over standard chemotherapy alone [92,93,94]. Even dual checkpoint blockade (e.g., nivolumab plus ipilimumab) yielded only incremental improvements in response and progression-free survival, benefiting a minority of patients while remaining limited by toxicity and lack of durability [95,96,97]. Collectively, these failures reflect fundamental features of ovarian cancer biology, including a predominantly “cold” TME, extensive stromal desmoplasia, low tumor mutational burden, and high expression of immunosuppressive pathways that limit T-cell infiltration and function [98,99,100,101,102,103]. The ECM and associated stromal components further contribute to immune exclusion and checkpoint resistance by physically restricting lymphocyte access and reinforcing immunosuppressive signaling networks. These effects reflect both mechanical exclusions, whereby dense and stiff matrices limit immune cell infiltration, and paracrine immunosuppression, mediated by TGF-β-driven signaling that dampens T-cell and macrophage effector functions, underscoring the rationale for combined stromal and immune-targeted interventions. Consequently, emerging therapeutic strategies increasingly emphasize TME normalization, including stromal and ECM remodeling and targeting immunosuppressive pathways such as TGF-β, to convert immune-excluded tumors into inflamed, ICI-responsive states.

TGF-β expression in the TME impedes the cytotoxic actions of tumor-infiltrating lymphocytes (TILs), as well as the tumoricidal actions of tumor-associated macrophages (TAMs) [104]. Hence, coordinated blockade of both TGF-β and immune checkpoints is of clinical interest. A proof-of-principle study showed that combinatorial blockade of TGF-β and programmed death-ligand 1 (PD-L1) using bintrafusp, the first bifunctional fusion complex consisting of human TGF-β receptor II fused to the heavy chain of an anti-PD-L1 IgG heavy chain elicited significant efficacy in the in vivo ID8 and BR5 OC models [105]. Bintrafusp treatment significantly reduced peritoneal tumor burden, extended survival, and impaired ascites development. Bintrafusp also depleted TGF-β, VEGF, and various inflammatory cytokines such as granulocyte colony-stimulating factor (G-CSF), IL-6, IL-5 and monocyte chemoattractant protein-1 (MCP-1), all of which are known drivers of OC metastasis. The TME was also shown to be significantly more inflammatory following bintrafusp treatment, with a marked increase in cytotoxic CD8 TILs and cytolytic natural killer (NK) cells, as well as increased expression of M1 TAM markers compared to controls [105]. Together, this study provides encouraging preclinical applicability of TGF-β inhibitors to normalize the OC TME to augment immunotherapy; however, the absence of validated biomarkers to guide dosing, scheduling, or patient selection warrants further investigation.

Despite these promising findings, the clinical translation of TGF-β inhibitors faces challenges. TGF-β plays context-dependent roles, acting as both a tumor suppressor and promoter [106]. TGF-β also influences multiple cellular and systemic functions, such as wound healing and immune homeostasis, which, when disrupted, may lead to off-target effects, including increased inflammation or immune evasion by tumors [76,107]. Furthermore, to date, there are no FDA-approved drugs that directly target TGF-β or its receptors. In summary, while TGF-β blockade effectively modulates tumor vasculature and lymphatics in HGSOC models and shows clear synergy with established immunotherapies and chemotherapies, translating these findings into clinical practice requires addressing its multifaceted roles, mitigating potential off-target effects, and being validated in preclinical models representing diverse histological and molecular subtypes of OCs.

## 4. Extracellular Matrix Normalization Through Losartan

Whilst TGF-β is an attractive target for TME normalization, its clinical translatability limits pragmatic implementation. Hence, we explored the use of losartan to modulate the tumor microenvironment and enhance the efficacy of chemotherapy in the HGSOC SKOV3ip1 and Hey-A8 OC models [25].

Losartan is an FDA-approved angiotensin II receptor blocker (ARB) that is approved for the treatment of hypertension in adults and children greater than 6 years old. Losartan is rapidly absorbed and undergoes extensive first-pass metabolism. The resulting active metabolite, EXP-3174, has a longer half-life and greater potency, accounting for the majority of the drug’s therapeutic effect [108]. In a preclinical breast cancer study, multiphysics modeling was employed to characterize losartan’s pharmacokinetics/pharmacodynamics, intratumoral penetration, and interstitial transport [109]. Simulating a standard 50 mg oral dose, the model demonstrated that the active metabolite has a half-life of ~6–9 h. The combined tissue concentration of losartan and EXP-3174 is predicted to remain above therapeutically relevant levels for approximately 24 h following a single dose [109]. In a pancreatic cancer mouse model, losartan was tested at various doses (10, 20, and 60 mg/kg). After 15 days of treatment, collagen production—assessed by second-harmonic generation imaging—was reduced by 20% (10 mg/kg), 33% (20 mg/kg), and 67% (60 mg/kg) [19].

The preclinical ovarian cancer study highlighted losartan’s ability to normalize the tumor stroma by reducing ECM content, particularly collagen. As previously discussed, the dense ECM in tumors contributes to high solid stress that compresses blood vessels, thereby hindering drug delivery. By decreasing the ECM content, losartan alleviated solid stress, leading to improved vascular perfusion and enhanced the delivery and efficacy of chemotherapy agents, such as paclitaxel, both intravenously and intraperitoneally [25]. This improvement is attributed to two key mechanisms: decompression of blood vessels and reduced ECM barriers that impede drug penetration. Critically, these effects are predominantly mechanical and vascular, targeting solid stress and perfusion rather than tumor-intrinsic signaling pathways, thereby distinguishing losartan-mediated ECM normalization from direct anti-fibrotic or anti-proliferative signaling inhibitors. Furthermore, these effects also translate into decreased tumor hypoxia, which can otherwise promote chemoresistance and tumor progression, as well as symptomatic alleviation of ascites. In the context of peritoneal dissemination, alleviation of solid stress and matrix density is expected to impair integrin-mediated spheroid adhesion to mesothelial surfaces and reduce the mechanical permissiveness required for metastatic niche formation, thereby disrupting early colonization events rather than initial spheroid survival.

**Figure 1:**
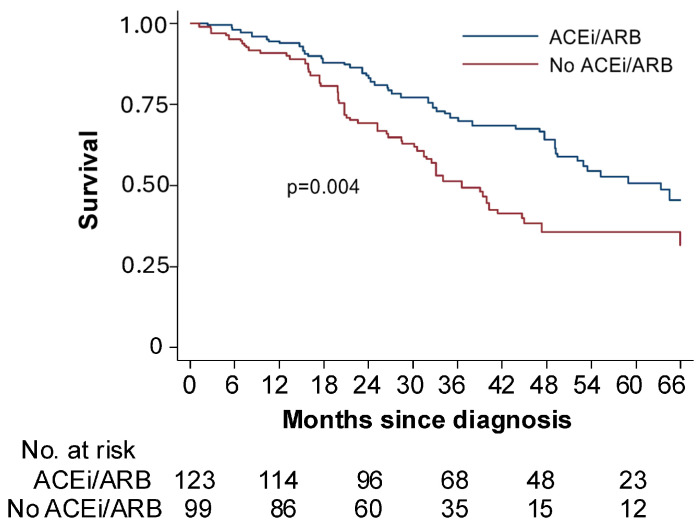
ACEi/ARB adjunctive treatment improves survival in women with ovarian cancer receiving standard of care. Retrospective analysis of patients with stage IIIC or IV ovarian cancer treated at Massachusetts General Hospital (MGH) and Brigham and Women’s Hospital (BWH) between 1 January 2010 and 31 December 2014. Women taking an angiotensin-converting enzyme inhibitors or angiotensin receptor blockers (ACEi/ARB; blue line) had a median survival of 63 months compared to 33 months among women taking another type of blood pressure medication (No ACEi/ARB; red line) at the time of cytoreductive surgery. Adapted from [25] with permission.

**Figure 2:**
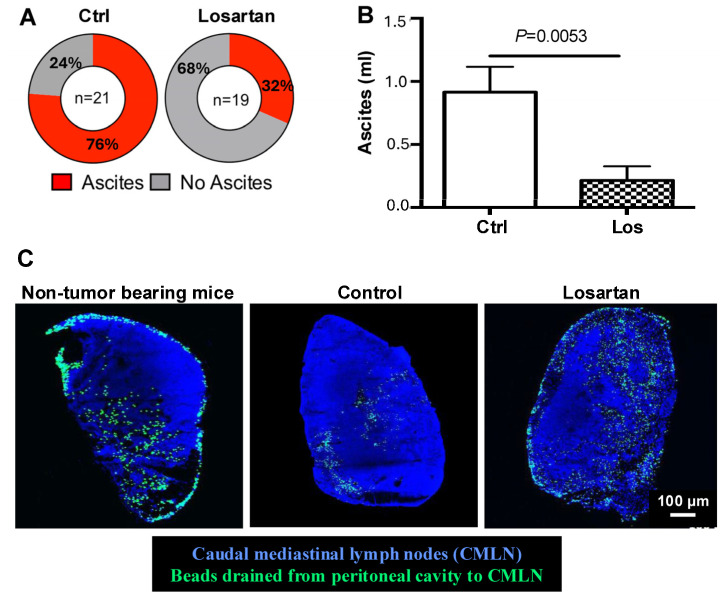
Losartan treatment reduces ascites via normalizing lymphatic vessel drainage function. (**A**) In the SKOV3ip1 model, which develops a significant amount of bloody ascites, losartan treatment significantly reduced the incidence and the amount of ascites. (**B**) Peritoneal fluid is drained by lymphatic vessels into the caudal mediastinal lymph nodes (CMLN). (**C**) To assess the lymphatic drainage function, we injected fluorescent beads intraperitoneally and measured their accumulation in CMLN. Compared with non-tumor bearing mice with normal drainage, CMLNs from SKOV3ip1 tumor-bearing showed reduced bead accumulation, indicating impaired drainage. Losartan treatment increased the number of fluorescence beads drained to CMLN, approaching levels seen in normal mice, demonstrating improved lymphatic drainage. Adapted from [25] with permission.

These findings were validated by retrospective analysis of patients with OC treated with angiotensin system inhibitors. Patients receiving these inhibitors alongside standard chemotherapy had overall survival exceeding 30 months, compared with those on other antihypertensives, underscoring losartan’s potential clinical benefits (Figure 1). The study also identified antifibrotic microRNAs (e.g., miR-133 and miR-29) that were upregulated by losartan and may serve as biomarkers of chemotherapy response and resistance in HGSOC models [25]. Thus, our study provides robust preclinical evidence and clinical translatability for incorporating losartan into OC treatment regimens. By normalizing the tumor microenvironment, losartan not only enhances chemotherapy efficacy, but also mitigates associated symptoms like ascites (Figure 2), presenting a promising strategy for improving OC patient outcomes and quality of life. Nevertheless, responses to renin–angiotensin system inhibition are unlikely to be uniform, as the magnitude of stromal normalization depends on baseline ECM density, vascular compression, and RAS pathway activity within individual tumors. This hypothesis is being prospectively tested in a Phase II clinical study evaluating the combination of losartan with weekly paclitaxel in patients with OC at MGH.

**Figure 3:**
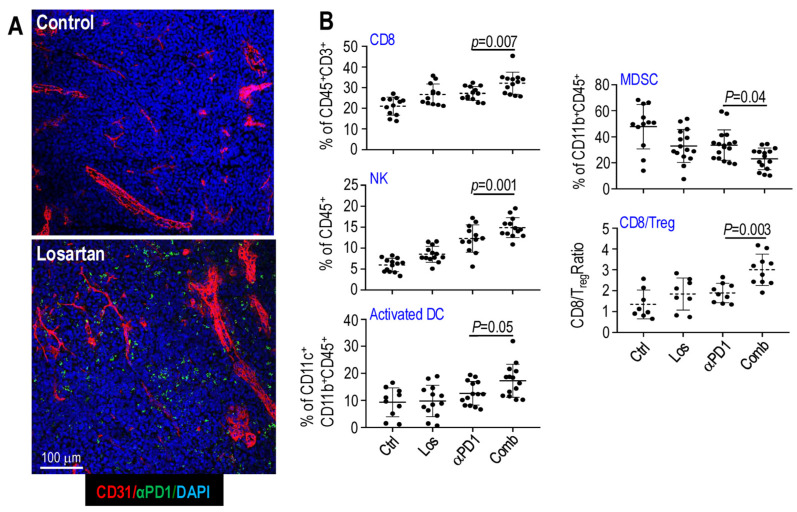
Losartan treatment enhances intratumoral drug delivery and immune effector cell infiltration in mouse OvCa models. (**A**) Losartan-treated tumors showed significantly increased intratumoral fluorescently labeled αPD1 antibody (green), indicating improved drug delivery. (**B**) Compared with αPD1 monotherapy, combined losartan treatment increased tumor-infiltration of CD8^+^ T cells, NK cells, and activated antigen-presenting dendritic cells (DCs), while reducing immune-suppressive MDSCs. The CD8/T_reg_ ratio was further elevated with the combination treatment. Adapted from [110] with permission.

Losartan’s ability to enhance the efficacy of chemotherapy warrants exploring whether it can also augment immunotherapy. Using two syngeneic BR5 and ID8 HGSOC OC models, we investigated the benefit of losartan in modulating the TME to enhance the efficacy of immunotherapy and anti-tumor immune responses in OC [110]. Our findings showed that losartan facilitated the delivery of ICIs, such as anti-PD-1 antibodies, into the TME (Figure 3A,B), thereby enhancing their efficacy. As demonstrated by the aforementioned study, losartan normalizes the TME by reducing ECM content and increasing vascular perfusion. This reprograms the immune microenvironment from immunosuppressive to immunostimulatory, as evidenced by elevated levels of intratumoral CD8^+^ T cells, NK cells, and activated dendritic cells, along with reduced suppressive Tregs and MDSCs (Figure 3B) [110]. These changes enhance the functionality of immune effector cells and cytokine production, further boosting anti-tumor immune responses. However, amplifying immune infiltration within previously immune-excluded tumors may also increase the risk of immune-related adverse events, particularly when combined with checkpoint blockade, highlighting the need for careful dose optimization and patient monitoring.

Losartan also directly suppresses insulin-like growth factor-1 (IGF-1) signaling within cancer cells. By inhibiting the IGF-1 pathway, losartan enhances chemosensitivity and reduces resistance to standard treatments like paclitaxel in the HGSOC models [110]. This is especially important given that elevated IGF-1 signaling is associated with chemotherapy resistance and poor outcomes in patients with OC [111,112]. Thus, losartan exerts multicompartmental effects, simultaneously acting on the vasculature (improved perfusion), immune compartment (enhanced immune infiltration and activation), and tumor cells (attenuated IGF-1 signaling), while indirectly reshaping CSC-supportive niches through ECM normalization. Together, our findings provide robust evidence supporting losartan’s utility in addressing the challenges posed by the abnormal TME and reversing treatment resistance to chemo-immunotherapy in patients with OC. Further studies should seek to identify and validate biomarkers to monitor losartan responsiveness for greater clinical utility, especially given the likelihood of heterogeneous responses across patients.

## 5. Conclusions

**Figure 4:**
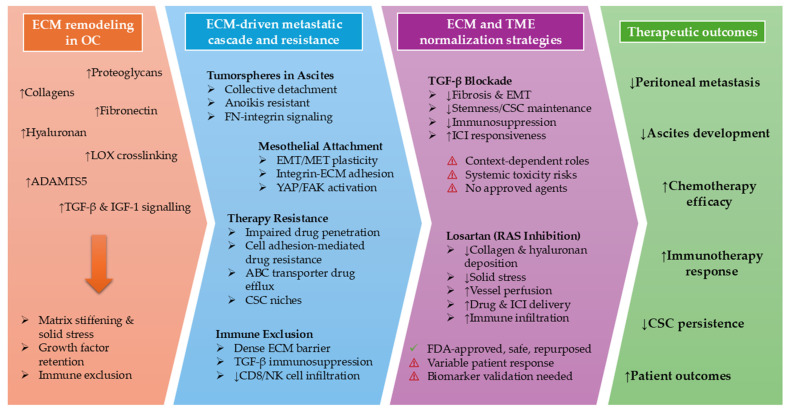
ECM remodeling drives peritoneal dissemination, metastatic colonization, and treatment resistance in ovarian cancer and is targetable through microenvironment normalization. OC progression is characterized by extensive remodeling of the ECM, including increased collagen deposition, fibronectin, hyaluronan, proteoglycans, and ECM-modifying enzymes, resulting in matrix stiffening, solid stress, and immune exclusion. These changes support tumorsphere survival in ascites, mesothelial attachment, EMT/MET plasticity, therapy resistance, and suppression of anti-tumor immunity. Therapeutic normalization strategies target these processes through distinct mechanisms: TGF-β blockade primarily modulates profibrotic and immunosuppressive signaling pathways, whereas losartan alleviates mechanical stress, improves vascular perfusion, and enhances drug and immune cell delivery. Together, ECM normalization disrupts metastatic colonization, restores treatment sensitivity, and improves therapeutic outcomes in patients with OC. NOTE: Prefix downward and upward facing arrows denote downregulation and upregulation, respectively.

Ovarian cancer remains a lethal gynecological malignancy, with late-stage diagnosis, extensive peritoneal dissemination, and high rates of relapse after first-line platinum–taxane chemotherapy continuing to limit long-term survival. Mounting evidence shows that these poor outcomes cannot be fully explained by tumor-intrinsic mechanisms alone but instead reflect profound abnormalities in the tumor microenvironment. In OC, various extracellular matrix (ECM) molecules drive metastasis and chemoresistance through multiple interconnected mechanisms (Figure 4).

- Collagens, particularly COL1 and COL6, are upregulated in primary and metastatic tumors, stiffen the matrix, enhance cell adhesion, and promote survival signaling, contributing to platinum and taxane resistance.
- Fibronectin interacts with integrins and L1CAM to support tumorsphere formation, EMT, anoikis resistance, mesothelial attachment, and activation of pro-survival Akt and survivin pathways, thereby facilitating invasion and chemoresistance.
- Proteoglycans, such as versican, form hydrated pericellular matrices in combination with hyaluronic acid that promote migration, adhesion, and resistance to mechanical stress while activating CD44-mediated signaling, ABC transporter expression, and stemness pathways like Nanog/STAT3, supporting multi-drug resistance.
- Heparan sulfate proteoglycans, including syndecans and perlecan, regulate growth factor bioavailability, cell–ECM adhesion, and drug penetration, sustaining pro-survival and angiogenic signaling under chemotherapy.
- ECM-remodeling enzymes such as ADAMTS5 and LOX mediate ECM cleavage, collagen crosslinking, and matrix stiffening, thereby promoting invasion, metastatic colonization, and chemoresistance.
- Laminin α5 supports proliferation and metastasis via Notch signaling, while interactions with adipocyte-derived factors and mesenchymal stem cells amplify ECM remodeling, EMT plasticity, and metastatic behavior.

Collectively, ECM composition, mechanical properties, and dynamic remodeling establish a protective microenvironment that promotes OC dissemination, survival, and resistance to chemotherapy. Importantly, these same ECM features also govern whether disseminated tumorspheres successfully transition into established peritoneal metastases, positioning ECM normalization as a strategy that selectively disrupts metastatic colonization rather than initial dissemination.

Targeting key upstream regulators of fibrosis and immunosuppression, therefore, offers a rational strategy to normalize the OC TME and restore treatment sensitivity. TGF-β sits at the center of this network, simultaneously driving collagen remodeling, EMT, stemness, angiogenesis, and immune exclusion. Preclinical studies in OC models demonstrate that blockade of TGF-β signaling reduces peritoneal tumor burden and ascites, improves vascular and lymphatic function, and enhances responses to chemotherapy. More recently, bifunctional agents that co-target TGF-β and PD-L1 have shown that simultaneous modulation of stromal and immune pathways can convert an immunosuppressive, fibrotic TME into an inflamed, therapy-responsive milieu, with increased cytotoxic T cells and NK cells. However, the pleiotropic and context-dependent roles of TGF-β in tissue homeostasis and early tumor suppression, together with the lack of approved TGF-β–targeted agents and concerns about systemic toxicity, currently constrain the immediate clinical deployment of direct TGF-β inhibition in OC.

In this context, tumor normalization using repurposed agents such as losartan represents a highly attractive and readily translatable approach. By targeting the renin–angiotensin system, losartan attenuates TGF-β-driven fibrotic signaling, reduces collagen and hyaluronan deposition, alleviates solid stress, and decompresses blood vessels, thereby improving perfusion, reducing hypoxia, and enhancing the intratumoral delivery and efficacy of both chemotherapy and immunotherapy in OC models. In parallel, losartan shifts the immune landscape towards an immunostimulatory state, increasing CD8^+^ T cells, NK cells, and activated dendritic cells, while limiting Tregs and MDSCs, and may further sensitize tumor cells by suppressing IGF-1-mediated survival pathways. Retrospective clinical data in patients with OC treated with angiotensin system inhibitors support these preclinical observations, suggesting improved survival when these agents are combined with standard chemotherapy.

Collectively, these findings position ECM remodeling and TME normalization at the center of future therapeutic strategies for OC (Figure 4). Rather than focusing solely on cytotoxic or tumor-intrinsic targeted therapies, integrating stromal- and immune-directed interventions offers a path to overcoming entrenched chemoresistance and improving patient outcomes. Whilst losartan is a highly promising new treatment strategy, there are still challenges to successful translation. The variability in tumor response to RAS inhibition highlights the need to identify biomarkers indicative of responsiveness, likely dependent on tumor characteristics and RAS component expression. Moreover, a greater understanding of the true therapeutic benefit of adding losartan to established cancer treatment regimes needs to be addressed through further preclinical testing and clinical trials. Efforts are underway at Massachusetts General Hospital to address these challenges, with a clinical trial planned to begin in 2026 to assess the efficacy of Losartan with chemotherapy to alleviate the symptomatic burden of ascites, identify clinically relevant biomarkers, and improve prognostic outcomes in patients with OC.

## Data Availability

No new data were created or analyzed in this study. Data sharing is not applicable to this article.

## Abbreviations

The following abbreviations are used in this manuscript:

ABC	ATP-binding cassette
ABCG1	ABC sub-family G member 1
ACE	Angiotensin-converting enzyme
ADAMTS5	A disintegrin and metalloproteinase with thrombospondin motifs 5
ALDH1A1	Aldehyde dehydrogenase 1A1
AngII	Angiotensin II
ARB	Angiotensin II type I receptor blocker
AT1	Angiotensin I
CAF	Cancer-associated fibroblast
CSPG	Chondroitin sulfate proteoglycan
CTR1	Copper transporter 1
ECM	Extracellular matrix
ECM1	Extracellular matrix protein 1
EGF	Epidermal growth factor
EMT	Epithelial-to-mesenchymal transition
FAK	Focal adhesion kinase
G-CSF	Granulocyte colony-stimulating factor
HIF	Hypoxia-inducible factor
HRD	Homologous recombination-deficient
HSGOC	High-grade serous ovarian cancer
HSPG	Heparan sulfate proteoglycan
ICI	Immune checkpoint inhibitor
IGF-1	Insulin-like growth factor-1
IL	Interleukin
L1CAM	L1 cell adhesion molecule
LOX	Lysyl oxidase
MCP-1	Monocyte chemoattractant protein-1
MDR1	Multi-drug resistance protein 1
MDSC	Myeloid-derived suppressor cell
MET	Mesenchymal-to-epithelial transition
NK	Natural killer cell
OC	Ovarian cancer
PARP	Poly ADP-ribose polymerase
PD-L1	Programmed death-ligand 1
PDGFRβ	Platelet-derived growth factor beta
RAS	Renin–angiotensin system
sTβRII	Soluble TGF-β receptor II
TAM	Tumor-associated macrophage
TGF-β	Transforming growth factor beta
TGFBR2	Type II TGF-β receptor
THBS-1	Thrombospondin-1
TIL	Tumor-infiltrating lymphocytes
TME	Tumor microenvironment
Treg	Regulatory T-cell
VEGF	Vascular endothelial growth factor
YAP	Yes-associated protein
